# Rongjin Niantong Fang ameliorates cartilage degeneration by regulating the SDF-1/CXCR4-p38MAPK signalling pathway

**DOI:** 10.1080/13880209.2022.2143533

**Published:** 2022-11-25

**Authors:** Jun Chen, Nan Chen, Ting Zhang, Jie Lin, Yunmei Huang, Guangwen Wu

**Affiliations:** a Academy of Integrative Medicine, Fujian University of Traditional Chinese Medicine, Fuzhou, China; b College of Integrative Medicine, Fujian University of Traditional Chinese Medicine, Fuzhou, China; c Key Laboratory of Orthopedics & Traumatology of Traditional Chinese Medicine and Rehabilitation (Fujian University of Traditional Chinese Medicine), Ministry of Education, China

**Keywords:** Osteoarthritis, chondrocyte, extracellular matrix, inflammation

## Abstract

**Context:**

Rongjin Niantong Fang (RJNTF) is a Traditional Chinese Medicine formulation with a good therapeutic effect on osteoarthritis (OA). However, the underlying mechanisms remain unclear.

**Objective:**

This study investigates whether RJNTF could delay OA cartilage degeneration by regulating the SDF-1/CXCR4-p38MAPK signalling pathway.

**Materials and methods:**

The Sprague-Dawley (SD) rats were used to establish the OA model by a modified Hulth’s method. SD rats were divided into three groups (*n* = 10): blank group, model group (0.9% saline, 10 mL/kg/day), and treatment group (RJNTF, 4.5 g/kg/day). After 12 weeks of treatment, each group was analysed by H&E, Safranine-O solid green, ELISA, Immunohistochemistry, and Western blot. An *in vitro* model was induced with 100 ng/mL SDF-1 by ELISA, the blank group, model group, RJNTF group, and inhibitor group with intervention for 12 h, each group was analysed by Immunofluorescence staining and Western blot.

**Results:**

SDF-1 content in the synovium was reduced in RJNTF treatment group compared to non-treatment model group (788.10 vs. 867.32 pg/mL) and down-regulation of CXCR4, MMP-3, MMP-9, MMP-13 protein expression, along with p38 protein phosphorylated were observed in RJNTF treatment group. *In vitro* results showed that RJNTF (IC_50_ = 8.925 mg/mL) intervention could down-regulate SDF-1 induced CXCR4 and p38 protein phosphorylated and reduce the synthesis of MMP-3, MMP-9, and MMP-13 proteins of chondrocytes from SD rat cartilage tissues.

**Discussion and conclusion:**

RJNTF alleviates OA cartilage damage by SDF-1/CXCR4-p38MAPK signalling pathway inhibition. Our ongoing research focuses on Whether RJNTF treats OA through alternative pathways.

## Introduction

Osteoarthritis (OA) is common in the middle-aged and elderly population, and the onset of the disease is mostly in the joints, especially in the knee joint, with clinical symptoms of pain, swelling, and causing inconvenience to the patient’s work and daily life and leading to disability in severe cases (Lin et al. 2017). The incidence of this disease increases with age. The onset of OA is mostly associated with factors such as gender, age, obesity, genetics, mechanical forces, and congenital abnormalities of the joint (Dobson et al. 2018). Under the combined effect of several factors, including mechanical and biological factors, degeneration of cartilage tissue occurs, the cartilage surface is roughened, and cartilage undergoes local to comprehensive destruction, eventually leading to the exposure of subchondral bone (Willy et al. 2008). Cartilage degeneration is a characteristic and fundamental pathological change in OA. Current clinical protocols for OA include oral NSAIDs, analgesics, condition-improving drugs (glucosamine, chondroitin sulfate), and surgical treatment (Glyn-Jones et al. 2015). However, certain limitations, such as gastrointestinal drug toxicity and cardiovascular adverse reactions remain in current clinical protocols (Tang et al. 2016). Effective prevention and treatment of OA remains a major challenge.

Specific binding of chemokine SDF-1 to CXC chemokine receptor family member CXCR4 exerts multiple biological effects. Experimental studies have shown that the binding of SDF-1 to CXCR4 leads to the degradation the of chondrocyte matrix by inducing the secretion of MMPs (Mazzetti et al. 2004; Lisignoli et al. 2006; Wei et al. 2006; Guang et al. 2012; Dong et al. 2016; Lu et al. 2016; Chen et al. 2017). As one of the important signalling pathways in eukaryotic cells, MAPK pathway regulates cell structure and function (Pawig et al. 2015). p38, an important component in the MAPK pathway, is closely related to cartilage degeneration OA and has the strongest effect on cartilage destruction. Activation of p38 can induce the secretion of chondrocyte MMPs, degrade the extracellular matrix, induce chondrocyte hypertrophy, mediate the inflammatory response, cell apoptosis, and other effects (Prasadam et al. 2012).

Rongjing Niantong Fang (RJNTF) originated from ‘Qing Gong Pei Fang Ji Cheng’ compiled by Academician Chen Keji (Chen. 2013). It consists of six herbs, *Achyranthes bidentata* Blume [Amaranthaceae], *Angelica sinensis* (Oliv.) Diels [Apiaceae], *Angelica biserrata* Radix [Apiaceae], *Hansenia weberbaueriana* (Fedde ex H.Wolff) Pimenov & Kljuykov [Apiaceae], *Saposhnikovia divaricata* (Turcz. ex Ledeb.) Schischk [Apiaceae], and *Glycyrrhiza uralensis* Fisch. ex DC [Fabaceae]. As a well-known traditional Chinese folk medicine, it is used to invigorate the circulation of swelling, relieve pain due to arthralgia, and tonify the liver and kidneys and the *Qi* and blood. It is commonly used for the treatment of various diseases, including knee osteoarthritis (KOA) (Zhu et al. 2017, 2020). However, it is unclear whether RJNTF can alleviate cartilage degeneration by regulating the SDF-1/CXCR4-p38MAPK signalling pathway. To further clarify the mechanism of action of this prescription, our study was conducted to observe CXCR4, p38 protein phosphorylation, MMP-3, MMP-9, and MMP-13 expression *in vivo* and *in vitro* to provide experimental evidence for the clinical application of RJNTF.

## Materials and methods

### Materials and reagents

RJNTF was provided by the Third People’s Hospital of Fujian University of Traditional Chinese Medicine (The National Invention Patent has authorised the preparation method and use of this formula, Patent No. ZL201710284659.8 and ZL201810899834.9). Pentobarbital sodium (3%) was purchased from Fuchen Chemical Reagents Factory (Tianjin, China). Paraformaldehyde (4%) was purchased from Yongda Chemical Reagent Co., Ltd. (Tianjin, China). The ELISA kit was obtained from Cloud-Clone Corp (Wuhan, China). An immunohistochemical kit was collected from Boster Bioengineering Co., Ltd. (Wuhan, China). The MMP-9 ELISA kit was purchased from R&D Systems (MN, USA). Type II collagenase solution (0.2%) was obtained from Sigma-Aldrich (MO, USA). Sodium penicillin was purchased from Hycione (USA). The primary antibodies against CXCR4 (ab124824), MMP-3 (ab25915), MMP-9 (ab38898), MMP-13 (ab39012), and p38 (ab170099) were purchased from Abcam (USA). The primary antibodies against Anti-rabbit IgG antibody (#14708), Anti-mouse IgG antibody (#4408), p-p38 (#4511), and GAPDH (#5174) were obtained from Cell Signalling Technology (USA). The antibody against vinculin (26520-1-AP) was purchased from Proteintech (USA). The antibody against CXCR4 (sc-53534) was obtained from Santa Cruz Biotechnology (USA).

### Animals

A total number of 30 male Sprague-Dawley (SD) rats (4-week-old, with a body weight of around 80 ± 10 g) and 30 male SD rats (8-week-old, with a body weight of around 200 ± 20 g) were purchased from SLAC Laboratory Animal Technology Co., Ltd. (Shanghai, China) (Certification NO. SCXK (Hu) 2017-0005, Shanghai, China). The rats were raised in the Experimental Animal Centre of Fujian University of Traditional Chinese Medicine (Certification No. SYXK (min) 2019-0007) under SPF conditions. The rats were housed in cages (five rats per cage) with free access to food and water. The environment was maintained at 24 ± 2 °C with a 12 h light/dark cycle. The experimental animal procedures were performed strictly according to international ethical guidelines and the National Institutes of Health Guide concerning the care and use of laboratory animals and were approved by the Institutional Animal Care and Use Committee of Fujian University of Traditional Chinese Medicine (Approval number: 2020053). The 8-week-old male SD rats were subjected to *in vivo* experiments, and the 4-week-old male SD rats were subjected to *in vitro* study to extract the primary chondrocytes.

### RJNTF preparation

RJNTF comprised of *Achyranthes bidentata, Angelica sinensis, Angelica biserrata, Hansenia weberbaueriana, Saposhnikovia divaricata*, and *Glycyrrhiza uralensis* in a ratio of 4:2:3:2:2:2. The decoction protocol of RJNTF is conducted as follows: add water according to the solid-liquid ratio of 1:10, decoct three times for 1.5 h each time, the liquid was filtered each time to remove the dregs and then combine and mix, evaporation with a rotary evaporator to lyophilise into powder, storage the lyophilised powder in a vacuum drying oven. For *in vivo* experiment, the equivalent dose for rats is six times the adult clinical dose (Huang et al. 2004), the adult clinical dose of Rongjin Niantong Fang is 45 g/60 kg, the equivalent dose of Rongjin Niantong Fang solution for rats = 45 g/60 kg × 6 = 4.5 g/kg. RJNTF was solubilised in double-distilled water to a final concentration of 0.45 g/mL and stored at −20 °C. Each rat was gavaged at a dose of 1 mL/100 g of body weight.

For *in vitro* experiments, RJNTF was extracted by reflux with the appropriate amount of distilled water at a solid-liquid ratio of 1:10 for three times for 1.5 h. The residue was removed by centrifugation, filtered, and the filtrate was combined and lyophilised into powder after rotary evaporation and concentration. RJNTF lyophilised powder was weighed 100 mg and added to a mother solution prepared with 1 mL of sterile PBS at a concentration of 100 mg/mL, it was filtered through a 0.22 μm filter tip and stored in a sealed container at 4 °C.

An ACQUITY UHPLC I-Class system coupled with a Xevo XS quadrupole time of flight mass spectrometer (Waters, Milford, MA, USA) was applied to fingerprint chemical components in RJNTF. Chromatographic separation was carried out at 45 °C on a Waters CORTECS C_18_ column (2.1 mm × 100 mm; 1.6 μm), with 0.1% of formic acid in water as mobile phase A and acetonitrile as mobile phase B. Gradient elution was performed as follows: 5–5% B for 0–0.5 min, 5–9% B for 0.5–1.5 min, 9–11% B for 1.5–4.5 min, 11–12.5% B for 4.5–6.5 min, 12.5% B for 6.5–9.5 min, 12.5–20% B for 9.5–10.5 min, 20% B for 10.5–17 min, 20–25% B for 17–22.5 min, 25% B for 22.5–27.5 min, 25–30% B for 27.5–32 min, 30% B for 32–37 min, 30–55% B at 37–39 min, and 55–90% B at 39–42 min. The flow rate was 0.25 mL/min. The mass-spectrometry conditions were optimised: ESI negative mode, dissolvent gas temperature, 500 °C; capillary voltage, 2.5 kV; source temperature, 150 °C; dissolvent gas flow, 800 L/h; and cone gas flow, 50 L/h. The MS scan range was m/z 50–1000.

### Grouping, model preparation, and processing

Thirty rats were randomly divided into the blank, model, and treatment groups (*n* = 10). After a week of routine feeding, rats were anaesthetised by intraperitoneal injection of 3% pentobarbital sodium (30 mg/kg). (Liu et al. 2005; Chen et al. 2016; Wu et al. 2019; Xu et al. 2021). Briefly, the rat model of OA was established using modified Hulth’s method in all groups except the blank group. A 1 cm longitudinal incision was made on the skin of the medial right-posterior knee, the medial collateral and anterior cruciate ligaments were transected *via* the medial approach, and the medial meniscus was removed. Then the joint capsule was sutured layer by layer. The blank group only received a 1 cm longitudinal incision on the skin of the medial right-posterior knee, and the skin was sutured. A prophylactic antibiotic with sodium penicillin (200,000 units) was given 3 days after surgery. The drawer test was used to determine whether the cruciate ligaments were transected.

Two weeks after the operation, a clinical oral dose of RJNTF (4.5 g/kg/day) was given to rats in the treatment group six times a week for 12 consecutive weeks. A dose of 0.9% saline (10 mL/kg/day) was simultaneously administered to the blank and model group.

### Specimen collection

After 12 weeks of intervention, rats were anaesthetised by intraperitoneal injection of 3% pentobarbital sodium according to 100 mg/kg. The knee joints of the rats were quickly opened, the synovial tissue was stripped and placed in liquid nitrogen, and the tibial plateau was separated. The general condition of the tibial plateau was immediately observed and recorded. The cartilage was placed in 4% paraformaldehyde for fixation at room temperature, and the tibial plateau was taken for an H&E staining, Safranine-O solid green, and immunohistochemical staining. Synovial tissues were collected for enzyme-linked immunosorbent assay (ELISA).

### H&E and Safranine-O solid green to explore the morphology of cartilage

The rat tibial plateau cartilage was cut into 5 × 4 × 3 mm sections, fixed in 4% paraformaldehyde for 2 days, and rinsed three times with running water. The tissue was decalcified in a decalcifying solution at room temperature and the decalcifying solution was replaced every 2 days for 8 weeks. Gradient ethanol dehydration and paraffin embedding were performed before sections were cut into 4 µm thick sections with a microtome. After dewaxed in xylene and dehydrated in ethanol H&E and Safranine-O solid green staining were performed. Cartilage morphology was observed *via* microscope, and images were obtained at a magnification of 100× or 200 ×.

### ELISA to detect SDF-1 content in the synovium

Synovial tissue (20 mg) was weighed and placed into a 1.5 mL tube with 1 mL of ice-cooled PBS. The synovial tissue was ultrasonically disrupted under ice-cooled conditions. Tissue suspension was centrifuged after vortex 10,000 rpm at 4 °C for 5 min, and the supernatant was pipetted into a 1.5 mL tube. The ELISA kit was taken out in advance and rewarmed at room temperature for 30 min. All sample locations were planned and arranged according to the number of holes in the plate coated with the ELISA kit. The standard product concentration was diluted according to the product instruction, the gradient concentration standard product was added and each group sample was according to the planned location, the detection solution A 100 μL was added according to the instruction, shocked evenly, pasted the sealing plate membrane, and incubated at 37 °C for 60 min. After incubation, the liquid was poured away and fully shaken, and then the washing solution was added to wash 3 times with a volley gun, 1–2 min each time. Finally, the washing solution was poured away. The detection solution B 100 μL was added to each well with a pipettor, pasted the sealing plate membrane, and incubated at 37 °C for 30 min. After incubation, the liquid was poured off and dried thoroughly. The washing solution was added 5 times with a volley gun, 1–2 min each time. Finally, the washing solution was poured out. The substrate solution of 90 μL was added to each well, the sealing plate film was pasted, and the stop solution of 50 μL was added to each well after 10 min at 37 °C to avoid light. The optical density (OD) was measured on a microplate reader at a wavelength of 450 nm (infinite M200 PRO, TECAN, Switzerland). According to the instruction method, the curve equation of the standard product and the SDF-1 concentration from the OD value of each hole was calculated.

### Immunohistochemistry analysis to detect the protein expression of CXCR4, MMP-3, MMP-9, and MMP-13 in cartilage

Each group’s rat tibial plateau cartilage specimens were cut into 5 × 4 × 3 mm sections, fixed in 4% paraformaldehyde for 2 days, decalcified in 10% EDTA for 8 weeks, dehydrated in gradient ethanol, and embedded in paraffin (Citotest Scientific, Jiangsu, China). The 5 µm thick paraffin sections were cut using a microtome, dewaxed in xylene, and analysed by immunohistochemistry. The sections were incubated with sodium citrate buffer (10 mM sodium citrate, pH 6.0) in a microwave for antigen retrieval. Endogenous peroxidase activity of the sections was quenched by incubating in PBS containing 0.3% H_2_O_2_ and 0.3% Triton X-100 for 30 min following repeated washing in PBS. According to the manufacturer’s instructions, immunohistochemical staining was performed using the test kit. Briefly, after blocking with normal serum in PBS, the sections were treated with an optimal dilution of primary antibody overnight at 4 °C. CXCR4 (1:500), MMP-3 (1:50), MMP-9 (1:100), and MMP-13 (1:100). The sections were incubated with a biotinylated anti-rabbit IgG antibody (1:1000) for 60 min and then treated with a reagent for 60 min. They were finally treated with DAB for 5 min. Subsequently, they were dehydrated with ascending concentrations of ethanol solutions, cleared with xylene, and mounted on a coverslip using neutral gum. The protein expression of CXCR4, MMP-3, MMP-9, and MMP-13 in cartilage tissue was photographed under a light microscope, and the protein expression was semi-quantitatively analyzed using Image-Pro Plus 5.1, and the protein expression levels were expressed as mean optical density (integrated density / area, IOD / area).

### Observation and identification of chondrocytes

Chondrocyte culturing and identification were performed as previously described (Lin et al. 2018). Briefly, knee joints of 4 weeks-old SD male rats were taken, and the cartilage tissues were isolated and cut into 1 mm^3^ piece, after PBS rinse, 4 mL 0.2% type II collagenase solution were added to digest in an incubator at 37 °C for 1.5 h. This digestion procedure was repeated three times. The digestive liquid was collected into a 15 mL centrifuge tube each time and centrifuged at 1000 rpm for 5 min, the supernatant was discarded, and 4 mL of cell culture media was added to resuspend the cell pellet, the cell suspension was then transferred to a 25 cm^2^ cell culture flask and incubated at 37 °C in a 5% CO_2_ incubator. 5 × 10^4^ second passed chondrocytes were inoculated on cell crawling piece and placed in six-well plates and cultured for 2 days then subjected to type II collagen immunocytochemical staining and toluidine blue staining, and observed and analysed in a light microscope (MDL, Leica, Germany).

### Cell counting kit-8 to detect the effect of RJNTF on chondrocyte viability

The second-generation chondrocyte suspension was inoculated in a 96-well plate and incubated overnight at 37 °C in an incubator, and the culture medium was discarded and replaced with 100, 300, 600, 1200, 2400, 4800, and 9600 μg/mL of RJNTF at different concentrations for 12 h. After incubation with 10 μL of CCK-8 reagent in each well for 2 h at 37 °C in an incubator under light-proof conditions, the OD was measured on a microplate reader at a wavelength of 450 nm by shaking for 20 s. And the value of IC_50_ was calculated.

### ELISA measurement of MMP-9

The second passage chondrocyte suspension was added to 24-well plates at a density of 1 × 10^5^/mL, 0.5 mL per well, and the intervention was performed after 48 h of culture. Different concentrations of SDF-1 (0, 50, 100, and 200 ng/mL) were used to intervene in rat chondrocytes, and the cell supernatants were taken in 1.5 mL tubes after 6, 12, and 24 h and labelled well. The ELISA kit was rewarmed at room temperature for 30 min, 50 μL RD1-34 was added to each well according to the planned layout of standards and samples, then 50 μL of gradient concentration standards and each group of samples respectively were added, and the sealing membrane was covered and incubated for 2 h at room temperature. The liquid was discarded and shaken off. Each well was filled with the washing solution and left to stand for 30 s, then the liquid was discarded and shaken off, and the washing was repeated 5 times. Rat Total MMP-9 Conjugate (100 μL) was added to each well, the sealing membrane was covered and incubated for 2 h at room temperature, and then the washing solution was repeated 5 times. Substrate solution (50 μL) was added to each well and incubated for 30 min at room temperature and avoided light, then 50 μL of stop solution was added to terminate the reaction. The OD was measured on a microplate reader (Promega, USA) at a wavelength of 450 nm. The equation of the standard curve was calculated according to the method in the instruction manual, and the concentration of MMP-9 was calculated according to the OD value of each well.

The second passage of chondrocyte suspension was added to 24-well plates at a density of 1 × 10^5^/mL, 0.5 mL per well, and the intervention was performed after 48 h of culture. The concentration and duration of SDF-1 induction were determined by the results of the ELISA experiment above. The cells were randomly divided into the blank group (regular culture), the model group (SDF-1 induction), the low-dose Chinese medicine group (SDF-1 + RJNTF 100 μg/mL), the medium-dose Chinese medicine group (SDF-1 + RJNTF 300 μg/mL), and the high-dose Chinese medicine group (SDF-1 + RJNTF 600 μg/mL). After 12 h of intervention, the cell supernatant was taken to calculate the concentration of MMP-9 by the ELISA method as above.

### Determine experimental grouping and intervention methods

The experimental groups were assigned as follows: Blank group, Model group, RJNTF group, and Inhibitor group with each intervention for 12 h as follows: Blank group: 10% FBS DMEM/LOW; Model group: 10% FBS DMEM/LOW containing 100 ng/mL SDF-1; RJNTF group: 10% FBS DMEM/LOW containing 100 ng/mL of SDF-1 and 300 μg/mL of RJNTF; Inhibitor group: 10% FBS DMEM/LOW containing 100 ng/mL SDF-1 as well as CXCR4 inhibitor AMD3100 5 ng/mL.

### Immunofluorescence observation of chondrocytes CXCR4 expression

The suspension of the second passage of chondrocyte was inoculated in laser-focused culture dishes at a density of 1 × 10^5^/mL, 1 mL per well, and the cells were cultured for 48 h before grouping for intervention. After the intervention, the supernatant was removed and the cell was gently rinsed three times with PBS. 500 μL of anhydrous methanol was added to each dish after the last rinse buffer was removed and left for 30 min at 4 °C. After three times PBS rinsing, 200 μL of 10% Triton was added and left for 10 min at room temperature to break the membrane. Following 3 times PBS rinsing, 200 μL 5% BSA (containing 10% foetal sheep serum) was added and blocked at room temperature for 1 h. The blocking solution was removed before adding 200 μL of diluted CXCR4 (1:250) antibody and incubated overnight for 4 °C. After three times PBS washing, 200 μL Anti-mouse IgG (1:1,000) was added and incubated at room temperature for 1 h. After three times PBS washing, 200 μL DAPI staining solution was added and incubated for 5 min at room temperature and protected from light. After three times PBS washing and sterilising three times, the CXCR4 protein expression was observed and recorded under a laser confocal microscope system.

### Western blot detection of CXCR4, MMP-3, MMP-9, and MMP-13 expression and p38 phosphorylation

The cartilage tissues were lysed with radioimmunoprecipitation analysis lysis buffer and phenylmethylsulfonyl fluoride on ice for 30 min to extract proteins. The BCA kit was applied to determine the protein concentrations of the sample. Protein samples were separated by SDS-PAGE and transferred to polyvinylidene fluoride membranes. The membranes were blocked with a blocking buffer at room temperature for 1 h. Then the membranes were incubated with antibodies against CXCR4 (1:100), p38 (1:1000), p-p38 (1:1000), MMP-3 (1:1000), MMP-9 (1:1000), MMP-13 (1:3000), vinculin (1:1000), and GAPDH (1:1000), respectively, overnight at 4 °C, then incubated with secondary antibody (1:1000) at room temperature for 1 h. Protein expression was quantified with BeyoECL Plus and analysed by ChemiDoc XRS + system (Bio-Rad, CA).

### Statistical methods

SPSS 24.0 software was used for statistical analysis. The measurement data were expressed in mean ± standard deviation (X ± s). The LSD method of one-way ANOVA was used to compare measurement data groups in line with normal distribution and homogeneity of variance. One-way ANOVA Games-Howell was used to compare groups for measurement data conformed to a normal distribution and variance disparity. The rank-sum test was used to compare groups for measurement data that did not conform to normal distribution. *p* < 0.05 means the difference is statistically significant.

## Results

### The medicinal chemical composition of RJNTF determined by UPLC-QTOF-MS model identification

**Figure 1.**
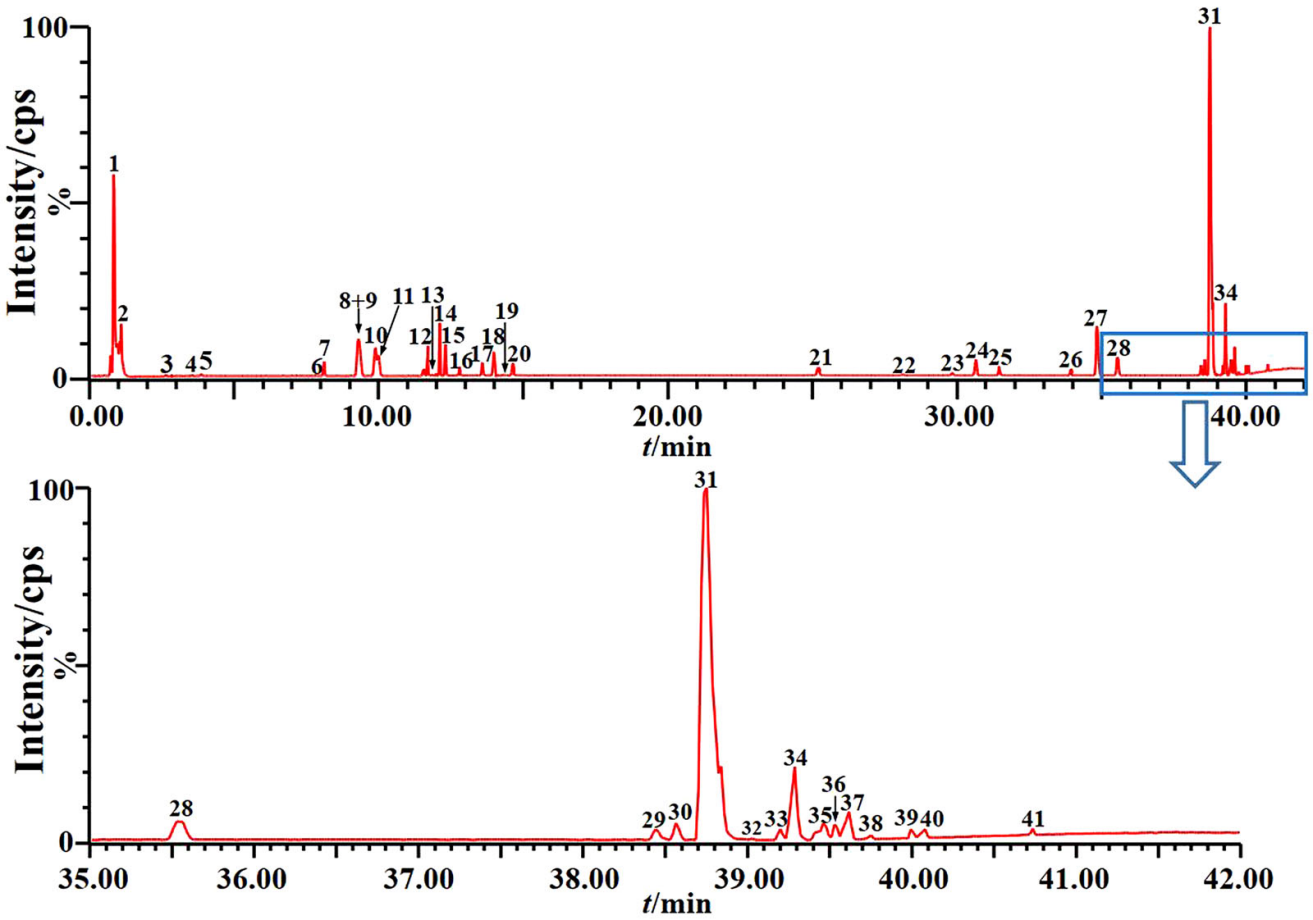
Total ion chromatogram fingerprint of RJNTF formulae.

A prescription amount of RJNTF was taken for analysis to obtain the total ion flow diagram in the negative ion mode of RJNTF (Figure 1). A total of 41 chemical components of RJNTF were identified using the precise molecular weights of individual components obtained by Q-TOF concerning the mass spectrometric information in the literature data, as shown in Table 1.

**Table 1.** Characterisation of chemical components in RJNTF by UPLC–QTOF–MS.

*t*_R_ (min)	Formula	Molecular ion (MS^1^)	Error (ppm)	Identification	Source
0.83	C12H22O11	341.1060 [M-H]-	7.04	Gentiobiose	G
1.10	C6H8O7	191.0176 [M-H]-	−8.38	Citric Acid	AS
2.64	C16H18O9	353.0860 [M-H]^-^	3.68	Neochlorogenic acid	G、H、AS
3.57	C16H18O9	353.0860 [M-H]-	3.68	Chlorogenic acid	G、H、AS
3.86	C16H18O9	353.0860 [M-H]-	3.68	Cryptochlorogenic acid	G、H、AS
7.93	C10H10O4	193.0484 [M-H]-	8.81	Ferulic acid	AS、AN
8.12	C_22_H_28_O_11_	467.1553 [M-H]^-^	4.28	Prim-*O*-Glucosylcimifugin	S
9.29	C_26_H_30_O_13_	549.1592 [M-H]-	−2.9	Liquirtin apioside	G
9.30	C_21_H_22_O_9_	417.1183 [M-H]^-^	0.72	Liquirtin	G
9.89	C_26_H_30_O_13_	549.1592 [M-H]-	2.91	Naringenin-7-*O*-(2-*β*-*D*-apiofuranosyl)-*β*-*D*-glucopyranoside	G
10.00	C_26_H_30_O_13_	549.1592 [M-H]-	−2.9	Liquirtin apioside isomer	G
11.70	C_27_H_44_O_7_	479.3003 [M-H]-	1.25	Ecdysterone	AC
11.99	C_27_H_44_O_7_	479.3003 [M-H]-	1.25	Inokosterone	AC
12.10	C_20_H_24_O_9_	407.1347 [M-H]-	−1.23	Nodakenin	H
12.32	C_22_H_28_O_10_	451.1607 [M-H]-	−0.66	5-*O*-Methylvisammioside	S
12.79	C_14_H_14_O_4_	245.0799 [M-H]-	6.12	Nodakenetin	H
13.60	C_26_H_30_O_13_	549.1592 [M-H]-	2.91	Isoliquiritin apioside	G
13.97	C_21_H_22_O_9_	417.1183 [M-H]-	−0.7	Isoliquiritin	G
14.27	C_22_H_22_O_9_	475.1212	−5.9	7-Methoxy-liquiritin	G
14.63	C_21_H_22_O_9_	417.1183 [M-H]-	−0.7	Neoisoliquiritin	G
25.19	C_15_H_16_O_3_	243.1004 [M-H]^-^	6.93	Osthole	AN
28.12	C_16_H_12_O_4_	267.0642 [M-H]^-^	5.62	Formononetin	G
29.85	C_48_H_72_O_21_	983.4487 [M-H]^-^	0.10	Licoricesaponine A	G
30.66	C_44_H_64_O_18_	879.3980 [M-H]^-^	3.87	22-Acetoxyl-glycyrrhizin or isomer	G
31.46	C_42_H_62_O_18_	853.3868 [M-H]^-^	−1.17	22-Hydroxyl-Licoricesaponin G2	G
33.93	C_42_H_60_O_16_	819.3778 [M-H]^-^	3.05	Licoricesaponine E2	G
34.84	C_44_H_64_O_18_	879.3980 [M-H]^-^	3.87	22-Acetoxyl-glycyrrhizin or isomer	G
35.53	C_48_H_76_O_19_	955.4874 [M-H]^-^	3.04	Ginsenoside Ro	AC、G
38.44	C_42_H_62_O_17_	837.3882 [M-H]^-^	3.22	Licoricesaponine G2	G
38.56	C_48_H_72_O_21_	837.3882 [M-H]^-^	3.22	22-Hydroxyl-glycyrrhizin	G
38.75	C_42_H_62_O_16_	821.3976 [M-H]^-^	−1.95	Glycyrrhizic acid	G
39.03	C_42_H_64_O_15_	807.4150 [M-H]^-^	2.11	Licorice saponine B2	G
39.20	C_42_H_64_O_15_	807.4150 [M-H]^-^	2.11	22-Dehydroxyl-uralsaponin C	G
39.29	C_42_H_62_O_16_	821.3976 [M-H]^-^	−1.95	Licoricesaponin H2	G
39.46	C_16_H_14_O_4_	269.0791 [M-H]^-^	8.55	Isoimperatorin	H、AN
39.53	C_42_H_64_O_16_	823.4133 [M-H]^-^	−2.06	Uralsaponin C	G
39.62	C_21_H_22_O_5_	353.1373 [M-H]^-^	4.53	Notopterol	H
39.75	C_42_H_62_O_16_	821.3976 [M-H]^-^	1.9	Uralsaponin B	G
39.98	C_47_H_70_O_20_	953.4354 [M-H]^-^	2.94	Achyranthoside I	AC
40.03	C_19_H_22_O_3_	297.1522 [M-H]^-^	−10.43	7-Geranyloxycoumarin	H
40.74	C_19_H_20_O_5_	327.1238 [M-H]^-^	−1.83	Columbianadin	AN

*Note. t_R_*_:_ retention time; G: *Glycyrrhiza uralensis*; H: *Hansenia weberbaueriana*; AN: *Angelica biserrata*; AC: *Achyranthes bidentata*; S: *Saposhnikovia divaricata*; AS: *Angelica sinensis.*

### Morphological observation of cartilage

**Figure 2.**
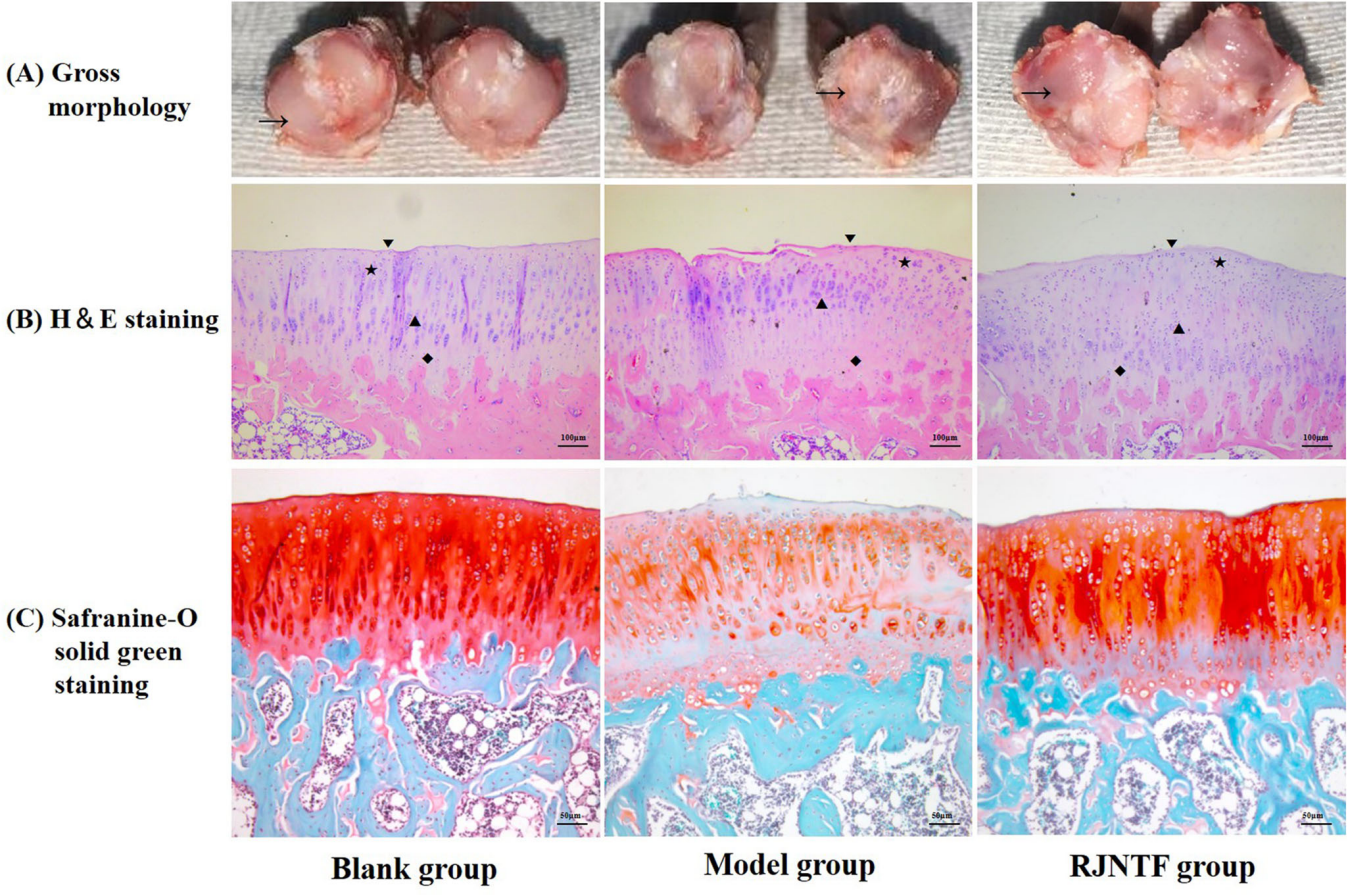
Morphological observation of cartilage. (A) a Gross morphology observation of cartilage in each group. The arrow marks the cartilage of the tibial plateau. (B) the H&E staining of cartilage tissue in each group. ▼: the superficial layer; ★: the transitional layer; ▲: the radiation layer; ◆: the calcified layer. (C) the Safranine-O solid green of cartilage tissue in each group.

Gross morphology observation showed that the cartilage surface of the tibial plateau of the joint of the rats in the blank group was flat, smooth, pink, and shiny. In the model group, the articular cartilage surface of rats was uneven and unsmooth, with morphological variation, bone redundancy at the edge of the plateau, reduction of cartilage in some areas, obvious local defects, and no lustre. The cartilage surface was slightly uneven with lustre in the treatment group, and bone flab formation was also seen at the platform’s edge (Figure 2).

H&E staining showed that the perichondrium of the knee joint of rats in the blank group was smooth, and the four layers of superficial layer, transitional layer, radiation layer, and the calcified layer of chondrocytes could be identified. In the model group, the cartilage surface was rough, there were fissures of different lengths, the fissures reached the superficial and transitional layer, the arrangement of chondrocytes was disorderly. Chondrocytes in the transitional layer and radiation layer showed hypertrophy and clustered. The articular cartilage surface in the treatment group was slightly rough, there were slight cracks in the local cartilage, the four-layer structure of cartilage tissue was slightly disordered. Compared with the model group, some chondrocytes in the transitional and radiation layers were clustered but not hypertrophic (Figure 2).

The cartilage tissue was evenly stained orange in the blank group, and the subchondral bone was green, indicating that the cartilage matrix contains the most proteoglycans. The staining of cartilage tissue in the model group was very light. The cartilage tissue was unevenly stained but darker in the treatment group than in the model group (Figure 2).

### Content of SDF-1 in the synovium

**Figure 3.**
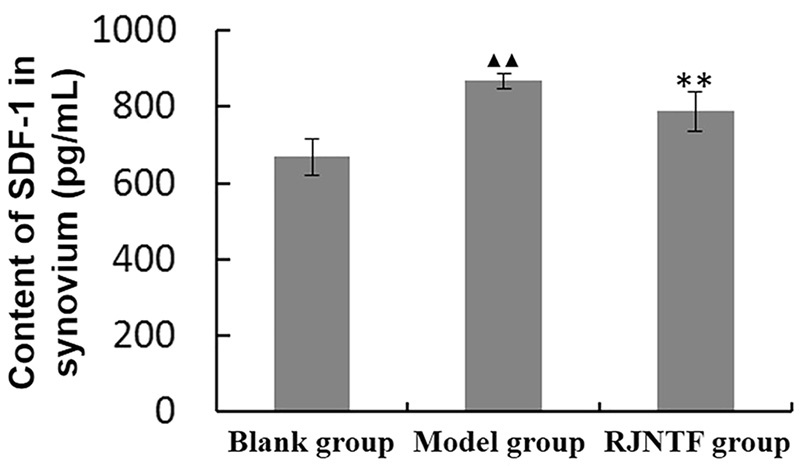
The content of SDF-1 in the synovium. RJNTF can reduce the content of SDF-1 in the synovium. Compared with the blank group, ^▲▲^*p* < 0.01; Compared with the model group, ^★★^*p* < 0.01.

As CXCR4 ligand SDF-1 binding to CXCR4 may induce MMPs secretion which leads to chondrocyte matrix degradation. Therefore, we were interested in the expression of SDF-1 in joint synovium. The result showed that amount of SDF-1 in the blank group was the least. The amount of SDF-1 in the synovium of the model group was significantly higher compared with the blank group. The amount of SDF-1 in the synovium of the treatment group was significantly lower compared with the model group, the difference was statistically significant (*p* < 0.01) (Figure 3).

### CXCR4, MMP-3, MMP-9, and MMP-13 protein expression in cartilage

**Figure 4.**
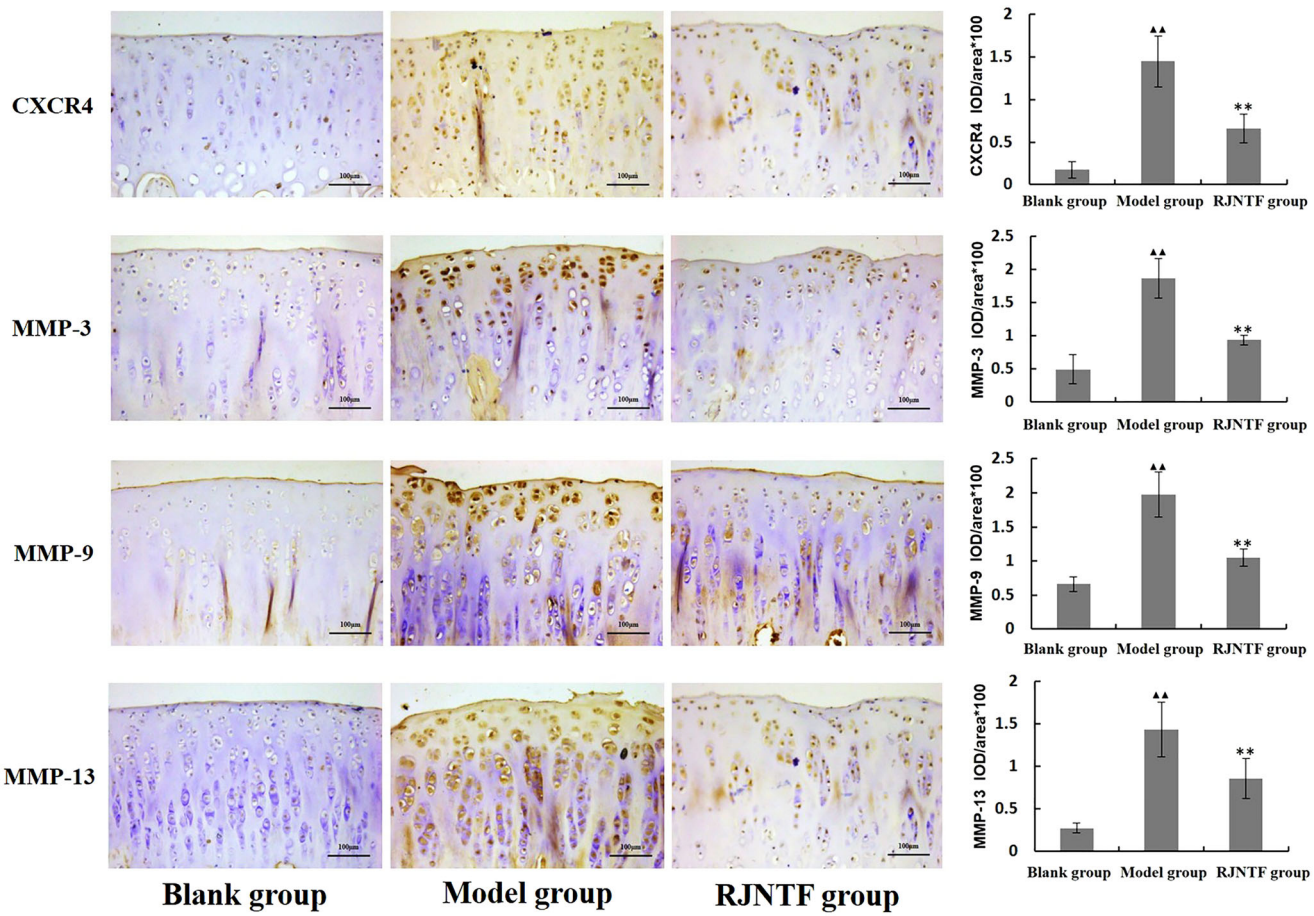
CXCR4, MMP-3, -9, -13 protein expression in cartilage tissue. RJNTF could reduce CXCR4 protein expression. Immunohistochemical maps of CXCR4, MMP-3, 9, and 13 in the blank, model, and RJNTF group, respectively (200×). ^▲▲^*p* < 0.01, ^★★^*p* < 0.01.

Since CXCR4 indicated the SDF-1 binding capacity, and matrix metalloproteinase family members MMP-3, MMP-9, and MMP-13 strongly related to chondrocyte matrix degradation. We investigated CXCR4, MMP-3, MMP-9, and MMP-13 expression in the joint cartilage of groups with different treatments. As shown in Figure 4, compared with the RJNTF treatment group, the expression of CXCR4, MMP-3, MMP-9, and MMP-13 in the cartilage tissue of the model group was higher, and the difference was statistically significant (*p* < 0.01).

### p38 phosphorylated protein expression in cartilage

**Figure 5.**
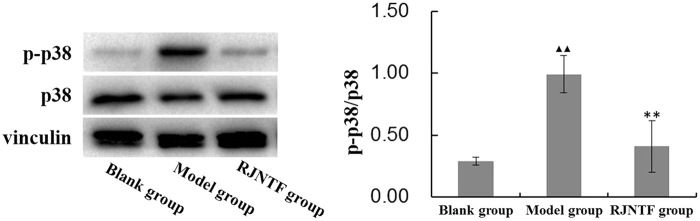
p38 phosphorylated protein expression in cartilage tissue. RJNTF could reduce p-p38 protein expression. ^▲▲^*p* < 0.01, ^★★^*p* < 0.01.

As an important effector protein in the MAPK signal transduction cascade, p38 would be phosphorylated *via* various stimulation including CXCR4 signalling. Phosphorylated p38 vs total p38 usually refers to the strength of the activation of the signal pathway. Therefore, we detected p38 phosphorylation of cartilage tissue of different treatment groups. The result showed that the ratio of phosphorylated p38 protein vs total p38 was significantly increased in the model group compared with the blank group. And the ratio of phosphorylated p38 protein vs total p38 decreased in the treatment group compared with the model group, the difference was statistically significant (*p* < 0.01 or *p* < 0.05) (Figure 5).

### Chondrocyte identification

**Figure 6.**
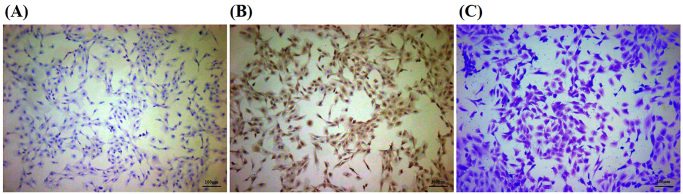
Chondrocyte identification (100×). (A) negative control for immunocytochemical staining of type II collagen. (B) immunocytochemical staining of type II collagen. (C) toluidine blue staining.

After the second passage, cultured chondrocytes were identified by Type II collagen immunocytochemical staining, and Toluidine blue staining. Results showed that in the negative control group’s nucleus of chondrocytes was stained blue, and the cell cytoplasm did not show brownish yellow (Figure 6(A)). Type II collagen immunocytochemical staining showed the cytoplasm of chondrocytes was brownish-yellow and the nucleus was blue (Figure 6(B)). Toluidine blue staining showed blue-purple staining in the chondrocyte cytoplasm (Figure 6(C)). All these referred that cultured cells could synthesise type II collagen, and proteoglycans and had chondrocyte functions which could be identified as chondrocytes.

### The viability of chondrocytes

**Figure 7.**
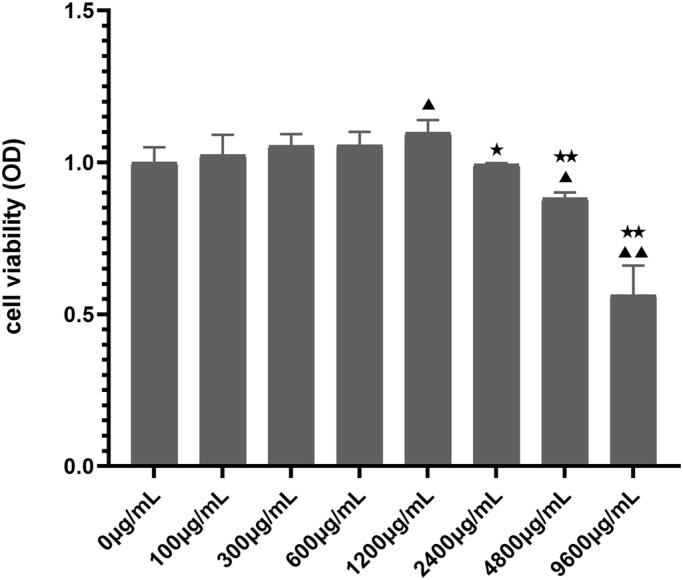
The effect of different concentrations of RJNTF intervention for 12 h in chondrocytes. Compared with 0 μg/mL, ^▲^*p* < 0.05, ^▲▲^*p* < 0.01, compared with 1200 μg/mL, ^★^*p* < 0.05, ^★★^*p* < 0.01.

The cell viability with RJNTF (concentration range, 0–600 μg/mL) showed no significant difference from the control group (Figure 7), indicating that RJNTF was not found to have an effect on cell viability until 600 μg/mL. The concentration of RJNTF at 1200 μg/mL showed a beneficial effect on cell viability (*p* < 0.05). And the cell viability was decreased at RJNTF concentration of 2400–9600 μg/mL (*p* < 0.05 or *p* < 0.01). The IC_50_ of RJNTF to the cell was at 8.925 mg/mL.

### The concentration and timing of SDF-1 induction in chondrocytes and the concentration of RJNTF in interfering with chondrocytes induced by SDF-1

**Figure 8.**
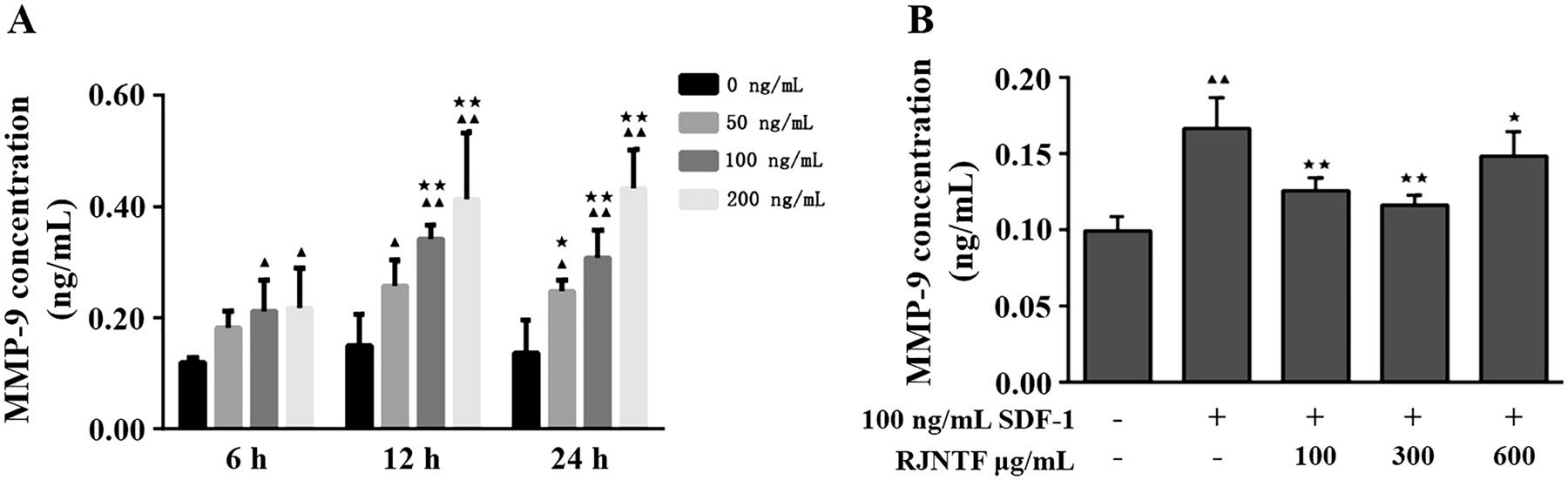
(A) Effective chronotropic efficacy diagram of SDF-1 intervention on chondrocytes. (B) the effective quantitative efficacy of RJNTF in interfering with SDF-1 induced chondrocytes. Same intervention time, compared to 0 ng/mL SDF-1, ^▲^*p* < 0.05, ^▲▲^*p* < 0.01; Same intervention concentration, compared to 6 h of intervention, ^★^*p* < 0.05, ^★★^*p* < 0.01. MMP-9 levels were lowest when intervening with RJNTF at 300 μg/mL concentration.

Different concentrations of SDF-1 intervention in chondrocytes after 6, 12, and 24 h were shown in (Figure 8(A)). SDF-1 (50, 100, and 200 ng/mL) promoted MMP-9 expression in chondrocytes at all time points compared with 0 ng/mL. At different time points, MMP-9 expression was elevated after 100 and 200 ng/mL SDF-1 intervention in chondrocytes compared with 0 ng/mL SDF-1 induction, with statistically significant differences (*p* < 0.05 or *p* < 0.01). 50, 100, and 200 ng/mL SDF-1 interventions in chondrocytes showed significantly higher amounts of MMP-9 content after 12 and 24 h interventions than after 6 h interventions (*p* < 0.05 or *p* < 0.01), and no significant difference in MMP-9 content after 12 and 24 h interventions. In conclusion, 100 ng/mL SDF-1 was chosen to induce chondrocytes at 12 h intervention to establish the model. Compared with the model group (Figure 8(B)), the MMP-9 content decreased after the intervention of 100, 300, and 600 μg/mL of RJNTF (*p* < 0.01 or *p* < 0.05), and the final concentration of RJNTF was determined to be 300 μg/mL.

### CXCR4 protein localisation in chondrocytes

**Figure 9.**
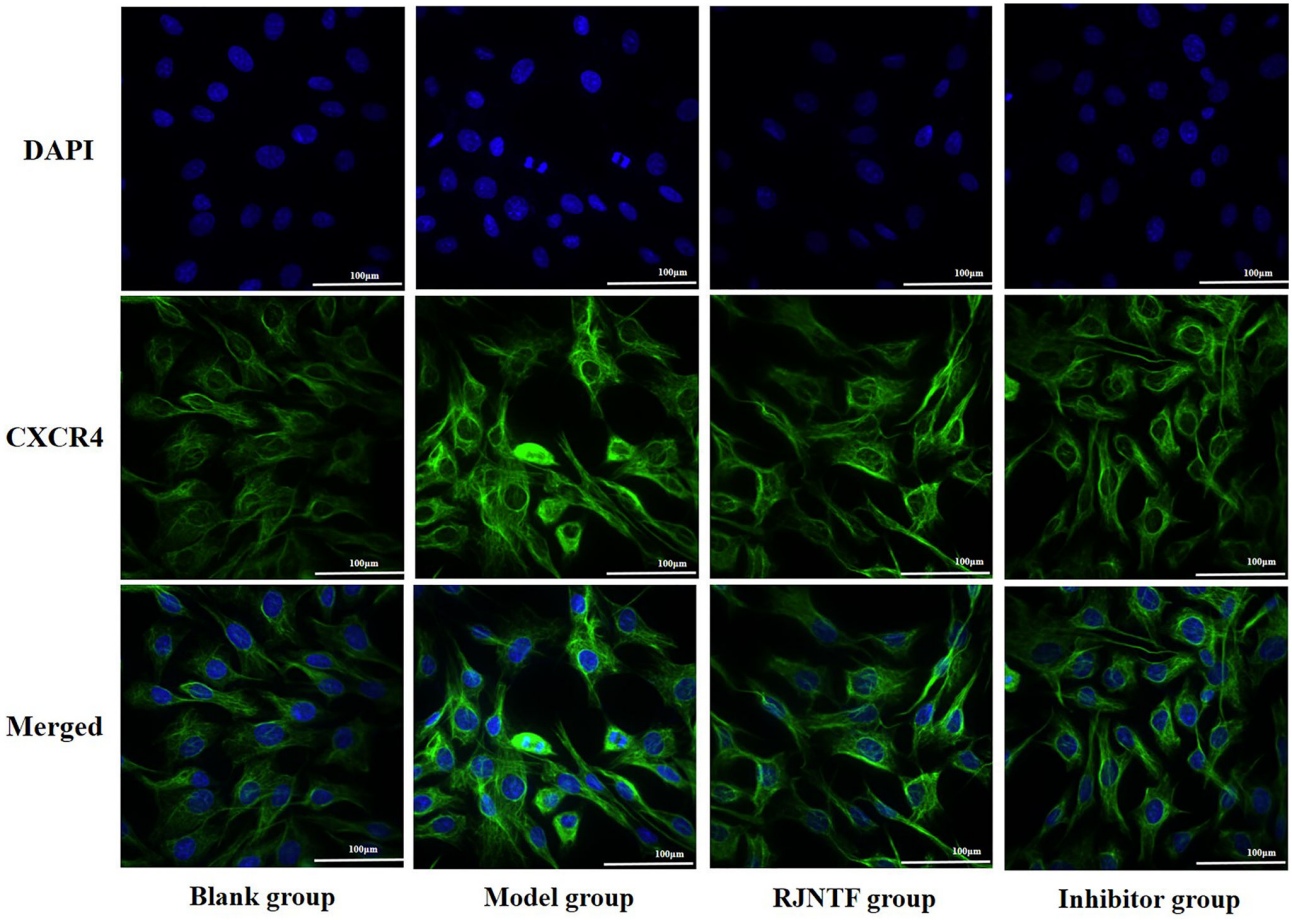
Laser confocal map of CXCR4 protein in each group of chondrocytes (400×). CXCR4 was intervened with RJNTF at a concentration of 300 μg/mL.

As shown in CXCR4 immunofluorescence staining (Figure 9), CXCR4 was positively expressed as green fluorescence. Compared with the blank group, the green fluorescence intensity in the model group was significantly enhanced. Compared with the model group, the green fluorescence intensity in the RJNTF and inhibitor group was significantly reduced.

### Protein expression of CXCR4, MMP-3, MMP-9, MMP-13, and p38 phosphorylation in chondrocytes

**Figure 10.**
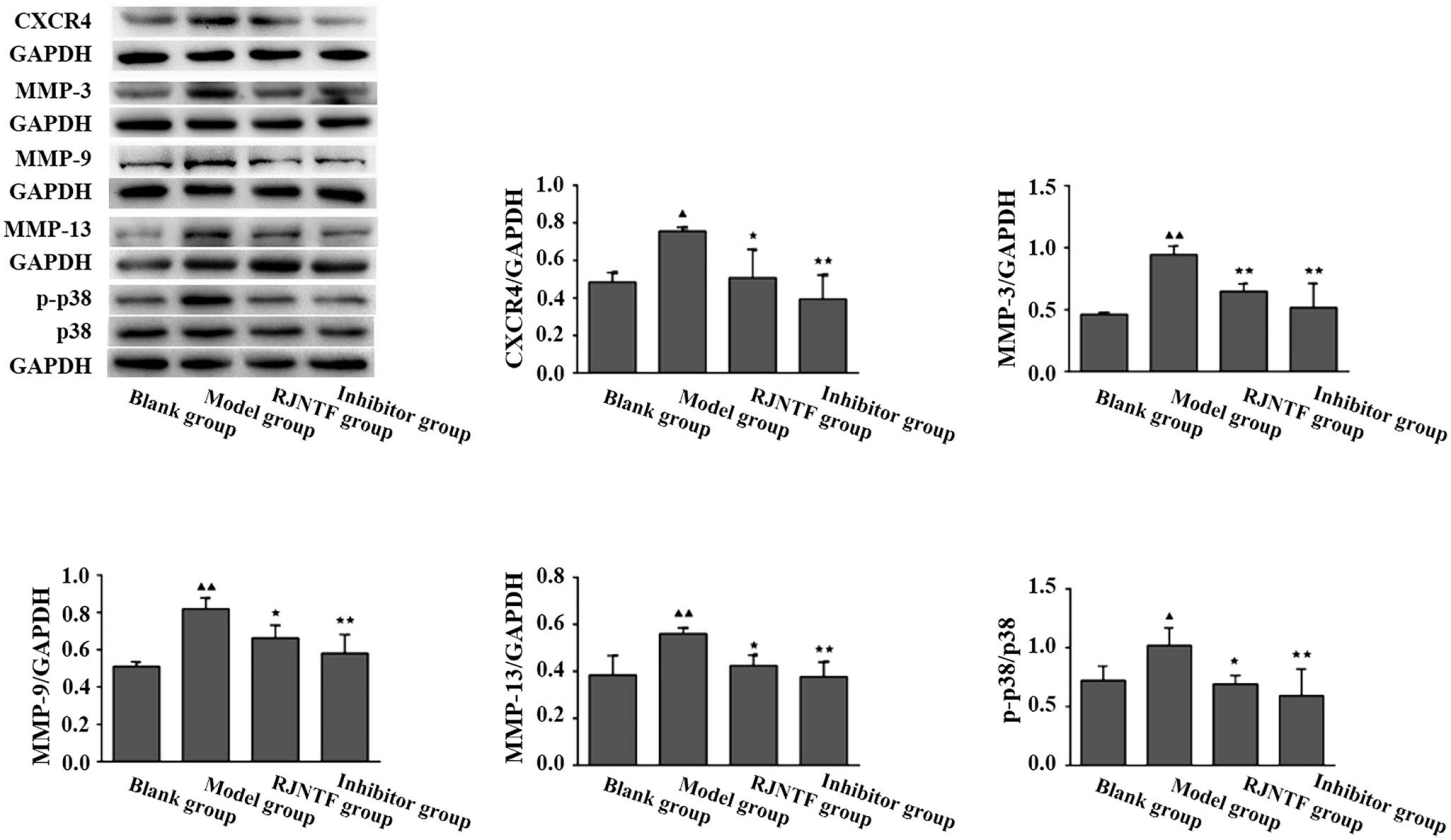
The protein expression of CXCR4, p38 phosphorylated, MMP-3, MMP-9, and MMP-13 in chondrocyte. ^▲^*p* < 0.05, ^▲▲^*p* < 0.01; ^★^*p* < 0.05, ^★★^ *p* < 0.01.

Immunoblotting results showed that the expression of CXCR4, MMP-3, MMP-9, MMP-13, and p38 protein phosphorylation was decreased in both RJNTF and the inhibitor group compared with the model group. The differences were statistically significant (*p* < 0.05 or *p* < 0.01) (Figure 10).

## Discussion

OA is a degenerative cartilage joint disease, one of the most common orthopaedic diseases, and multi-morbidity (Pereira et al. 2015). With the accelerated ageing process of our society, the incidence of OA has increased significantly, and how to effectively prevent and treat OA is a challenge for our future medical cause (Liu et al. 2018). More and more studies have shown that herbal formula plays a good therapeutic role in treating OA and has fewer side effects, which is worth exploring the mechanism. UPLC-QTOF-MS identifies 41 medicinal chemicals in RJNTF. Many studies have pointed out that the active ingredients of these herbal medicines play an important role in treating OA. For example, β-ecdysterone, the active ingredient of *Achyranthes bidentata,* might alleviate OA by activating autophagy in chondrocytes by regulating the PI3K/AKT/mTOR signal pathway (Tang et al. 2020). Ferulic acid in *Angelica sinensis* has antioxidant and anti-inflammatory characteristics and can scavenge free radicals, while *Angelica* polysaccharides can protect joint cartilage by promoting the expression of polyproteoglycans and glycosyltransferases. As the highest content of coumarin, the main component of *Angelica biserrata*, osthol has been shown to effectively reduce MMP-3, MMP-9, and MMP-13 levels in rat serum, suggesting that osthol can alleviate the degradation of rat cartilage matrix by reducing the level of MMPs (Xu et al. 2018). Notopterol is the most abundant component enriched by *Hansenia weberbaueriana*. Notopterol directly binds Janus kinase (JAK)2 and JAK3 kinase domains to inhibit JAK/signal transducers and activators of transcription (JAK-STAT) activation, leading to reduced production of inflammatory cytokines and chemokines (Wang et al. 2020). The prim-*O*-glucosylcimifugin (POG), from *Saposhnikovia divaricate,* has an anti-inflammatory effect on RAW 264.7 macrophages by inhibiting JAK2/STAT3 signalling and suppressing the expression of iNOS and COX-2 (Zhou et al. 2017). Glycyrrhizin, the active ingredient of *Glycyrrhiza uralensis*, has anti-allergic and anti-inflammatory functions. The six herbs help nourish the liver and kidney, strengthen the tendons and bones, invigorate the circulation of swelling, and relieve paralysis and pain, and their anti-inflammatory effects protect and promote the repair of joint cartilage, which provide a theoretical basis for the treatment of OA.

Accumulating evidence suggests that cartilage degeneration is a typical pathological manifestation of OA. Cartilage tissue comprises chondrocytes and an extracellular matrix containing collagen, proteoglycan, water, and inorganic salts (Schulz and Bader 2007). When the biomechanical equilibrium of the body is modified, disturbances in load transfer caused by changes in joint stress may lead to articular cartilage injury. Increased intra-articular pressure or leakage of water from cartilage tissues reduces the absorption of nutrients into the articular cartilage, resulting in necrosis of the articular cartilage and the production of factors such as proteases that break down collagen and proteoglycans, leading to the loss of cartilage matrix and promoting the destruction of articular cartilage. Therefore, protecting cartilage tissue and delaying the degeneration of articular cartilage play an important role in the prevention and treatment of OA. In this experiment, after the intervention, the cartilage in the model group showed OA cartilage tissue lesions under visual. After the intervention of RJNTF, the cartilage tissue structure improved, matrix staining deepened, cartilage cell structure and collagen fibre morphology improved, and cartilage degeneration in rats was reduced. It is suggested that RJNTF can inhibit the degradation of proteoglycans, prevent the loss of the cartilage matrix, maintain the balance of chondrocytes and extracellular matrix, and thus delay the degeneration of articular cartilage in rats.

In normal conditions, articular chondrocytes and extracellular matrix synthesis and degradation are dynamic equilibrium. When an imbalance disrupts the state, protease MMPs can degrade and destroy the extracellular matrix (Rai et al. 2019). MMP-3 is a zinc ion-dependent enzyme that degrades extracellular matrix components, and its overexpression promotes the disintegration of cartilage framework structures, which can be used as a reference index to assess the severity of OA (Li et al. 2013; Park et al. 2017). MMP-13 is strongly degraded in degenerated cartilage (Long and Loeser 2010) and promotes the production of MMP-9 to degrade extracellular matrix (Wang et al. 2019). In normal joints, the expression of MMPs is at a low level, while in OA, the expression of MMPs is greatly increased.

MMPs play a significant role in articular cartilage destruction. It can also activate and amplify the degradation ability of the cartilage matrix and aggravate the damage of articular cartilage in OA. Therefore, we believe that up-regulation (down-regulation) of MMP-3, MMP-9, and MMP-13 can be used as an 'indicator’ of activation (suppression) of the SDF-1/CXCR4-p38MAPK signalling pathway. In this experiment, the expression of MMP-3, MMP-9, and MMP-13 in cartilage tissues of the rats’ model of OA was up-regulated, while the expression of MMP-3, MMP-9, and MMP-13 in the treatment group with RJNTF intervention was significantly reduced, suggesting that RJNTF can effectively inhibit the secretion of MMPs and thus prevent the degradation of extracellular matrix.

Notably, what regulates the secretion of MMPs? It was found that activation of the MAPK signalling pathway promotes the secretion of MMPs downstream of the regulatory pathway, while the p38MAPK signalling pathway stimulates apoptosis in chondrocytes and accelerates chondrocytes hypertrophy and calcification (Li et al. 2015). When the p38MAPK pathway is activated, it promotes the secretion of MMPs, degrading the extracellular matrix and destroying collagen and proteoglycans (Liacini et al. 2003). Related studies have shown that after the surgical method of establishing OA models in rats or rabbits, cartilage degenerates gradually, p38 phosphorylation protein expression increases in degenerated cartilage, and periodic tension stresses of different strengths acting on normal cartilage for several weeks also cause phosphorylation of p38 (Huang et al. 2008). Some studies found that after the injection of p38 inhibitor SB203580 into the joint cavity of osteoarthritic rats, the expression of MMP-3, MMP-9, and MMP-13 in cartilage was downregulated, chondrocyte apoptosis was reduced, and cartilage degeneration became relieved (Chen et al. 2007; Yan et al. 2020). Therefore, we believe that inhibition of chondrocyte p38 activation is beneficial in reducing the expression of MMPs, thereby alleviating the degeneration of articular cartilage. In this experiment, the expression of p38 phosphorylated protein in cartilage tissues was reduced in the treatment group, suggesting that RJNTF can regulate the p38MAPK signalling pathway to inhibit the secretion of downstream MMPs and protect the extracellular matrix.

SDF-1 is a CXC-like chemokine, and its expression is increased in the synovial membrane of osteoarthritis. It was found that the level of SDF-1 in the joint fluid of osteoarthritis patients was 3.57 times higher than that of normal people, and the level of SDF-1 after synovectomy was 5.1 times lower than before surgery (Kanbe et al. 2002, 2004). SDF-1 has been reported to penetrate articular cartilage, suggesting that SDF-1 synthesised by synovial cells can freely diffuse into adjacent cartilage (Fermas et al. 2008). A complementary expression pattern exists between SDF-1 and CXCR4 (Möhle et al. 1998), with synovial membrane producing SDF-1 and its receptor CXCR4 preferentially expressed by articular chondrocytes, and CXCR4 expression positively correlated with cartilage degeneration (Chen et al. 2011). CXCR4 binds to SDF-1 to form the SDF-1/CXCR4 signalling pathway, which regulates OA chondrocyte development (Wei et al. 2012). Wei et al. (2006) found that higher than 200 ng/mL SDF-1 induced human chondrocyte necrosis and that fewer chondrocytes died after anti-CXCR4 treatment. Nanki et al. (2009), Qin et al. (2019), and Zhang et al. (2022) found that the SDF-1/CXCR4 pathway was involved in maintaining or promoting the inflammatory process in KOA patients and that inhibition of the SDF-1/CXCR4 pathway slowed the development of OA and promoted knee recovery. CXCR4 specifically recognises and then forms a complex with SDF-1, which induces increased expression of MMPs through the SDF-1/CXCR4 signalling pathway, leading to cartilage matrix degradation and cartilage structural destruction, thus participating in the regulation of OA disease progression (Melgarejo et al. 2009). In this experiment, SDF-1 content in the synovial membrane and cartilage CXCR4 protein content was reduced after intervention with RJNTF in the treatment group, consistent with the results reported above, indicating that RJNTF can reduce the expression of the downstream proteins MMP-3, MMP-9 and MMP-13 by regulating the SDF-1/CXCR4-p38MAPK signalling pathway, maintaining the dynamic balance of chondrocytes and extracellular matrix, and reducing the structural damage of cartilage and the destruction of cartilage structure.

*In vitro* experiments were undertaken using SDF-1 to intervene in chondrocytes to validate further. The aim is to simulate the stimulation model of chondrocytes by high expression of SDF-1 in the joint fluid of OA. Chondrocytes induced by SDF-1 showed increased expression of CXCR4, elevated expression of p38 phosphorylated protein of MAPK pathway, and elevated expression of MMP-3, MMP-9, and MMP-13. RJNTF can reduce the expression of CXCR4, p38 phosphorylated protein of MAPK pathway and MMP-3, MMP-9, and MMP-13 after the intervention. RJNTF inhibits the synthesis of MMPs in OA chondrocytes by regulating the SDF-1/CXCR4-p38MAPK signalling pathway and delays the degeneration of rat chondrocytes to treat OA. This experiment explored the mechanism of action of RJNTF that can treat OA and provide an experimental basis for the clinical treatment of OA with RJNTF. The relationship between the SDF-1/CXCR4-p38MAPK signalling axis and OA was also further investigated to provide new ideas for the treatment of OA. In addition, OA is characterised by cartilage degeneration, changes in subchondral bone, and secondary synovial joint inflammation, which influence each other in OA progression (Primorac et al. 2020). Studies have found that inhibiting the SDF-1 signalling with AMD3100 could reduce the aberrant subchondral bone formation in the rat OA model, and it showed that SDF-1 plays an important role in subchondral bone in OA development (Chen et al. 2017). Therefore, we will design experiments to investigate whether RJNTF can regulate subchondral bone *via* SDF-1 to treat OA and provide a theoretical basis for further elucidating the pathogenesis of OA.

## Conclusions

The present study showed that RJNTF could delay OA cartilage degeneration by regulating the SDF-1/CXCR4-p38MAPK signalling pathway using the rat’s cartilage tissue with OA *in vivo* and SDF-1 induction of extracorporeal chondrocytes *in vitro* experiments. Hence, we suggest that the application of RJNTF could be a new strategy for OA therapy.

## Data Availability

The original contributions presented in the study are included in the article. Further inquiries can be directed to the corresponding authors.
